# Correction to: ‘A triple threat: high population density, high foraging intensity and flexible habitat preferences explain high impact of feral cats on prey’ (2021) by Hamer *et al.*

**DOI:** 10.1098/rspb.2022.1985

**Published:** 2022-11-30

**Authors:** Rowena P. Hamer, Riana Z. Gardiner, Kirstin M. Proft, Christopher N. Johnson, Menna E. Jones

**Affiliations:** ^1^ School of Natural Sciences, University of Tasmania, Hobart, Tasmania 7005, Australia; ^2^ Tasmanian Land Conservancy, Hobart, Tasmania 7005, Australia

*Proc. R. Soc. B* **288**, 20201194 (Published 13 January 2021) (https://doi.org/10.1098/rspb.2020.1194)

Since publication, an error has been discovered in the code used to calculate the population densities of spotted-tailed quolls and feral cats in Hamer *et al*. [1], results from which were used to calculate rates of encounter in this study [2]. Correcting this error changes the magnitude of differences between the encounter rate of prey with feral cats and spotted-tailed quolls, but does not change the direction or interpretation of these results. In correcting these results, we also encountered a typographical error, where the estimated average density was reported rather than encounter rate of cats and quolls in the text. Below, we highlight changes to the manuscript text in bold type, and present the updated figures 1 and 3. We have also updated and replaced electronic supplementary material, appendix S1.

**Figure 1.**
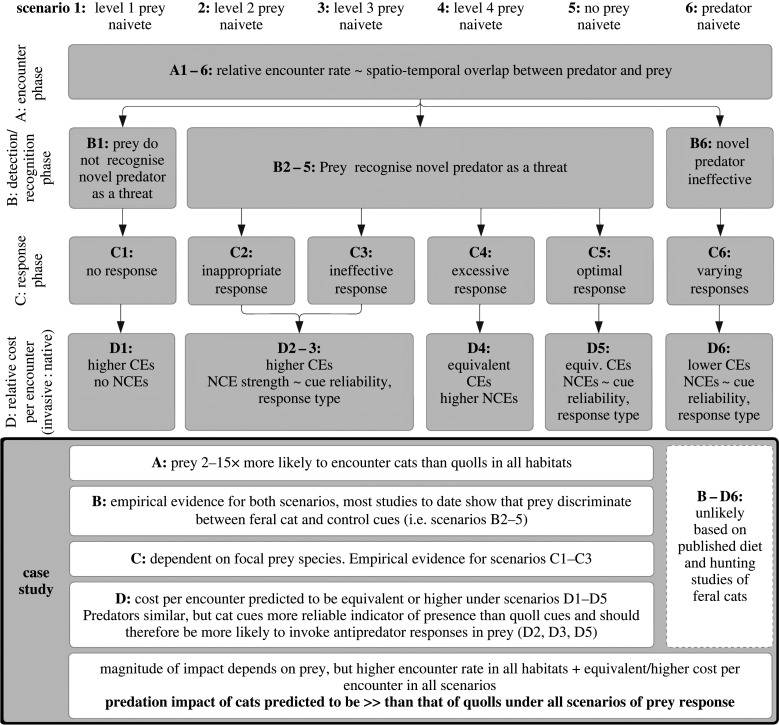
Conceptual framework for assessing relative predation impact of native versus introduced predators on shared native prey species. Predation impact depends on the rate of encounter between predator and prey (A), and the costs associated with each encounter (D). For our case study, rather than estimate the impact of each predator on a focal prey species, we predict the relative impact of cats versus quolls on all shared prey. These predictions are based on theoretical classes of prey response (scenarios 1 : 6, derived from prey naivete theory [2,3]) during the stages of a predator-prey encounter (stages A : C, [8,9]), to estimate the relative strengths of both consumptive and non-consumptive effects (D, [5,6]).

**Figure 3.**
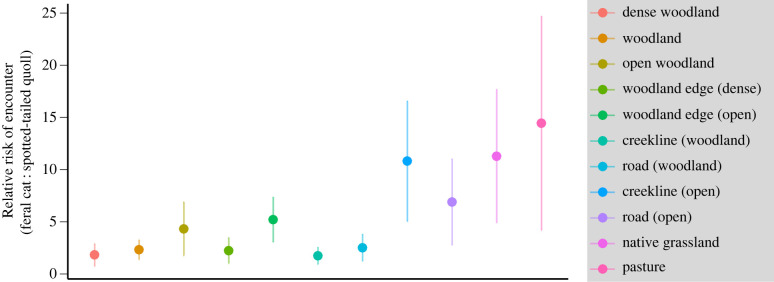
Relative risk of encountering feral cats and spotted-tailed quolls in different habitat types across the Tasmanian Midlands landscape. (Online version in colour.)

Abstract

Alien mammalian carnivores have contributed disproportionately to global loss of biodiversity. In Australia, predation by the feral cat and red fox is one of the most significant causes of the decline of native vertebrates. To discover why cats have greater impacts on prey than native predators, we compared the ecology of the feral cat to a marsupial counterpart, the spotted-tailed quoll. Individual prey are **2–15** times more likely to encounter feral cats, because of the combined effects of cats' higher population densities, greater intensity of home-range use and broader habitat preferences. These characteristics also mean that the costs to the prey of adopting anti-predator behaviours against feral cats are likely to be much higher than adopting such behaviours in response to spotted-tailed quolls, due to the reliability and ubiquity of feral cat cues. These results help explain the devastating impacts of cats on wildlife in Australia and other parts of the world.

Results

(a) Risk of encounter

Across the Midlands landscape, the risk of encountering a cat was always at least **twice** the risk of encountering a quoll (figure 3). On average, the probability of a cat being present within each 30 m raster cell on any one night was **0.00016 ± 0.00004**, compared to **0.00004 ± 0.00001** for quolls (electronic supplementary material, figure S1.4).

(b) Population density and revisitation frequency

Cats had consistently higher population densities than quolls in the Midlands (averaging approximately **0.7** cats km^−2^ versus 0.4 quolls ha^−1^, electronic supplementary material, table S1.2). Cats also revisited areas within their home ranges almost twice as frequently as quolls (cats 8.0 ± 0.6 visits month^−1^ cell^−1^, versus quolls 3.5 ± 0.3 visits month^−1^ cell^−1^, electronic supplementary material, figure S1.1).

Discussion

(a) Risk of encounter

While the absolute rate of encounter will vary according to the habitat preferences and behaviour of prey species, native prey are between approximately **2–15** times more likely to encounter a cat than a quoll in all habitats across our study area (figure 3). A large proportion of this difference is due to differences in density. Average densities of cats in the Midlands (**0.65** cats km^−2^ [18]) are higher than the national average of 0.27 cats km^−2^ [24].

## Data Availability

Supplementary material is available online [3].
